# SIRT3 and SIRT5 Regulate the Enzyme Activity and Cardiolipin Binding of Very Long-Chain Acyl-CoA Dehydrogenase

**DOI:** 10.1371/journal.pone.0122297

**Published:** 2015-03-26

**Authors:** Yuxun Zhang, Sivakama S. Bharathi, Matthew J. Rardin, Radha Uppala, Eric Verdin, Bradford W. Gibson, Eric S. Goetzman

**Affiliations:** 1 Department of Pediatrics, University of Pittsburgh, Children's Hospital of Pittsburgh, Pittsburgh, Pennsylvania, United States of America; 2 Buck Institute for Research on Aging, Novato, California, United States of America; 3 Gladstone Institutes, University of California San Francisco, San Francisco, California, United States of America; Nihon University School of Medicine, JAPAN

## Abstract

SIRT3 and SIRT5 have been shown to regulate mitochondrial fatty acid oxidation but the molecular mechanisms behind the regulation are lacking. Here, we demonstrate that SIRT3 and SIRT5 both target human very long-chain acyl-CoA dehydrogenase (VLCAD), a key fatty acid oxidation enzyme. SIRT3 deacetylates and SIRT5 desuccinylates K299 which serves to stabilize the essential FAD cofactor in the active site. Further, we show that VLCAD binds strongly to cardiolipin and isolated mitochondrial membranes via a domain near the C-terminus containing lysines K482, K492, and K507. Acetylation or succinylation of these residues eliminates binding of VLCAD to cardiolipin. SIRT3 deacetylates K507 while SIRT5 desuccinylates K482, K492, and K507. Sirtuin deacylation of recombinant VLCAD rescues membrane binding. Endogenous VLCAD from SIRT3 and SIRT5 knockout mouse liver shows reduced binding to cardiolipin. Thus, SIRT3 and SIRT5 promote fatty acid oxidation by converging upon VLCAD to promote its activity and membrane localization. Regulation of cardiolipin binding by reversible lysine acylation is a novel mechanism that is predicted to extrapolate to other metabolic proteins that localize to the inner mitochondrial membrane.

## Introduction

Mitochondrial fatty acid β-oxidation (FAO) is a critical energy-producing pathway in humans. Heart, muscle, and liver oxidize large amounts of fatty acids for energy and the function of these organs becomes severely compromised in patients with inborn errors of FAO [1]. Dysregulation of FAO has also been implicated in the pathophysiology of common disorders such as diabetes, obesity, and cancer [2–4]. One mechanism contributing to FAO dysregulation in such diseases may be alterations in protein acylation and sirtuin function. SIRT3 and SIRT5 are both thought to positively regulate FAO as evidenced by reduced flux through the pathway in knockout mice [5, 6]. The reduced FAO flux is concomitant with increased lysine acylation on FAO proteins—acetylation in SIRT3-/- mice and succinylation in SIRT5-/- mice—suggesting that acylation is deleterious to the FAO machinery. Proteomics studies of mouse tissues and human cell lines indicate that the post-translational landscape of FAO proteins is complex, with virtually every enzyme in the FAO pathway being subject to acylation on multiple lysines [5, 7–9].

We previously used chemical hyper-acetylation combined with targeted deacetylation to elucidate the SIRT3-targeted lysines on the FAO enzyme long-chain acyl-CoA dehydrogenase (LCAD)[10]. Of the 15 lysines known to be acetylated on LCAD *in vivo*, SIRT3 was found to specifically target two residues near the active site. LCAD is abundantly expressed in rodents and this mechanism likely contributes to the reduced FAO flux observed in SIRT3 knockout mice. In humans, however, LCAD expression is absent from critical FAO-dependent tissues such as heart and muscle [11, 12]. Rather, very long-chain acyl-CoA dehydrogenase (VLCAD) predominates. LCAD and VLCAD have largely overlapping substrate specificities and both use electron transferring flavoprotein (ETF) as an electron acceptor, but they are structurally different. LCAD is a homotetramer localized to the mitochondrial matrix while VLCAD is a homodimer that interacts peripherally with the inner mitochondrial membrane. The importance of proper VLCAD localization is highlighted by the presence of disease symptoms in patients with missense mutations in the putative membrane-binding domain, despite normal enzyme activity when assayed *in vitro* [13].

VLCAD’s partners in long-chain FAO are carnitine palmitoyltransferase-2 (CPT2) and mitochondrial trifunctional protein (TFP). Both are peripheral membrane proteins that have been shown to bind to cardiolipin [14, 15]. The importance of cardiolipin for promoting FAO is reflected in the observation that the rate of FAO flux correlates with cardiolipin content across different tissue types [16]. Cardiolipin has structural importance in mitochondrial membranes, helping to form contact sites between the inner and outer mitochondrial membranes and serving as the platform for the assembly of respiratory chain supercomplexes [17]. FAO proteins associate with respiratory chain supercomplexes and with each other in order to facilitate efficient substrate channeling and re-oxidation of the NADH and FADH_2_ produced by FAO [18]. We now show that VLCAD is also a cardiolipin-binding protein, and that acylation of three lysines in the membrane-binding domain can regulate the association of VLCAD with cardiolipin. SIRT3 and SIRT5 both target this domain to facilitate cardiolipin binding. This mechanism is postulated to affect other FAO proteins and perhaps regulate the formation of metabolic supercomplexes on the inner mitochondrial membrane.

## Materials and Methods

### Mouse breeding and tissue isolation

Breeding of VLCAD-/-, SIRT3-/-, and SIRT5-/- mice and collection of tissues was approved by the University of Pittsburgh Institutional Animal Care and Use Committee (IACUC Protocol #12090974). Euthanasia was conducted using inhaled CO2 gas according to IACUC recommendations. Mice were fasted overnight (16 hr) prior to euthanasia and harvesting of the liver for use in blotting experiments or mitochondrial isolation.

### Protein expression and purification

The expression vector for human SIRT5 was developed by Cheryl Arrowsmith (Addgene plasmid 25487). The recombinant expression system for human SIRT3 was previously described [6]. For human VLCAD, a 6-histidine tag was introduced at the amino terminus of the VLCAD isoform 2 cDNA (NM 001033859). Mutagenesis of VLCAD was done using the QuickChange II Site-Directed Mutagenesis kit (Agilent Technologies). All His-tagged proteins were purified on HisTrap^TM^HP columns using an AKTA-FPLC system (GE Healthcare Life Sciences).

### Chemical acylation

Chemical acetylation was performed with sulfo-NHS-acetate (Thermo Scientific, Rockford, IL) as described [10]. Succinylation was done with sulfo-NHS-succinate which was prepared as follows. 9.7 mg of sodium succinate, 7.8 mg of NHS, and 9.7 mg of EDC were dissolved in 300 μl of dimethylformamide in a glass vial and incubated at room temperature for 16 hr. From this reaction, 50μl was removed to a new vial, dried under nitrogen, and washed with ether. After re-drying, the material was dissolved in 50 μl of water and incubated immediately with VLCAD protein for 2 hr at room temperature. All acylated proteins were dialyzed overnight prior to experimentation.

### VLCAD activity assays

The anaerobic electron transfer flavoprotein (ETF) fluorescence reduction assay was performed as described [10]. Briefly, 200 ng of protein samples and 1 μM purified porcine ETF were mixed in a sealed, degassed cuvette at 32°C. The decrease in ETF fluorescence was followed after adding palmitoyl-CoA substrate to 25 μM (Sigma, St. Louis, MO). Specific activity was calculated from the slope and Y-intercept and expressed as milliunits (mU) of activity per mg of protein.

### Western blotting

VLCAD was detected using either mouse monoclonal anti-poly-His antibody (Sigma, St. Louis, MO) at a 1:5000 dilution or rabbit anti-VLCAD antiserum at 1:2500. Lysine acetylation and succinylation were detected using rabbit anti-acetyllysine antibody (Cell Signaling Technology, Beverly, MA) and anti-succinyllysine antibodies [5], respectively, both at 1:1000 dilution. After incubation with HRP-conjugated secondary antibodies (1:5000) blots were visualized with chemiluminescence.

### Membrane binding

Mouse liver mitochondrial membranes were prepared from VLCAD knockout mice as described [13], and 4.5 μg of mitochondrial membranes were incubated with 200 ng of recombinant VLCAD protein in 210 mM mannitol, 70 mM sucrose, 5 mM HEPES, pH 8.0 at 37°C for 90 minutes. Reactions were separated into supernatant and membrane pellet fractions by centrifugation at 21,000 x g. In some experiments, multilamellar vesicles were prepared and used for VLCAD binding. An 8:1 mixture of phosphatidylcholine: cardiolipin in chloroform was dried to produce a lipid film which was then rehydrated at 52°C for 5 hr. After cooling to room temperature, VLCAD was added and incubated for 1 hr at 37°C, followed by centrifugation at 78,000 x g for 20 minutes. Fat blot experiments were done as described [19].

### Sirtuin deacylation

Deacylation reactions were performed as described for SIRT3 [10] and SIRT5 [7]. In some experiments deacylation was followed as the cleavage of ^14^C-NAD^+^ (Perkin Elmer, Waltham, MA) to ^14^C-NAM as described [10] with a reaction time of 20 minutes at 37°C.

### Sirtuin target site identification

Quadruplicate samples of chemically-acylated VLCAD were treated with either active SIRT3/SIRT5 or inactive mutant SIRT3/SIRT5 as controls. Reverse-phase LC-ESI-MS/MS analysis was done in duplicate with an Eksigent Ultra Plus nano-LC 2D HPLC system (Dublin, CA) connected to a quadrupole time-of-flight TripleTOF 5600 mass spectrometer (AB SCIEX) in direct injection mode as previously described [5]. Peptides were identified using the ProteinPilot search engine with the following sample parameters: trypsin digestion, cysteine alkylation set to iodoacetamide, urea denaturation, and succinylation or acetylation emphasis. Peptides were searched against the Swiss-Prot entry P49748, and peptides were chosen based on a 95% confidence level followed by manual inspection. Raw MS data files were imported into Skyline and precursor ion chromatograms were extracted for label free quantification using MS1 Filtering as previously described [20]. Peptide areas were then averaged across all sample acquisitions and a ratio generated.

### Protein molecular modeling

Three-dimensional modeling of the VLCAD enzyme (PDB 3B96) was performed with Yasara software (Yasara Biosciences, Vienna, Austria), except for the putative membrane binding helix which was modeled with PSIPRED v3.3.

### Statistical analyses

Statistics were performed using the Student’s *t*-test in Microsoft Excel. Curve-fitting for the NAD hydrolysis assay was performed in Graphpad Prism 6.0.

## Results

### SIRT3 and SIRT5 have overlapping target sites on VLCAD

**Fig 1:**
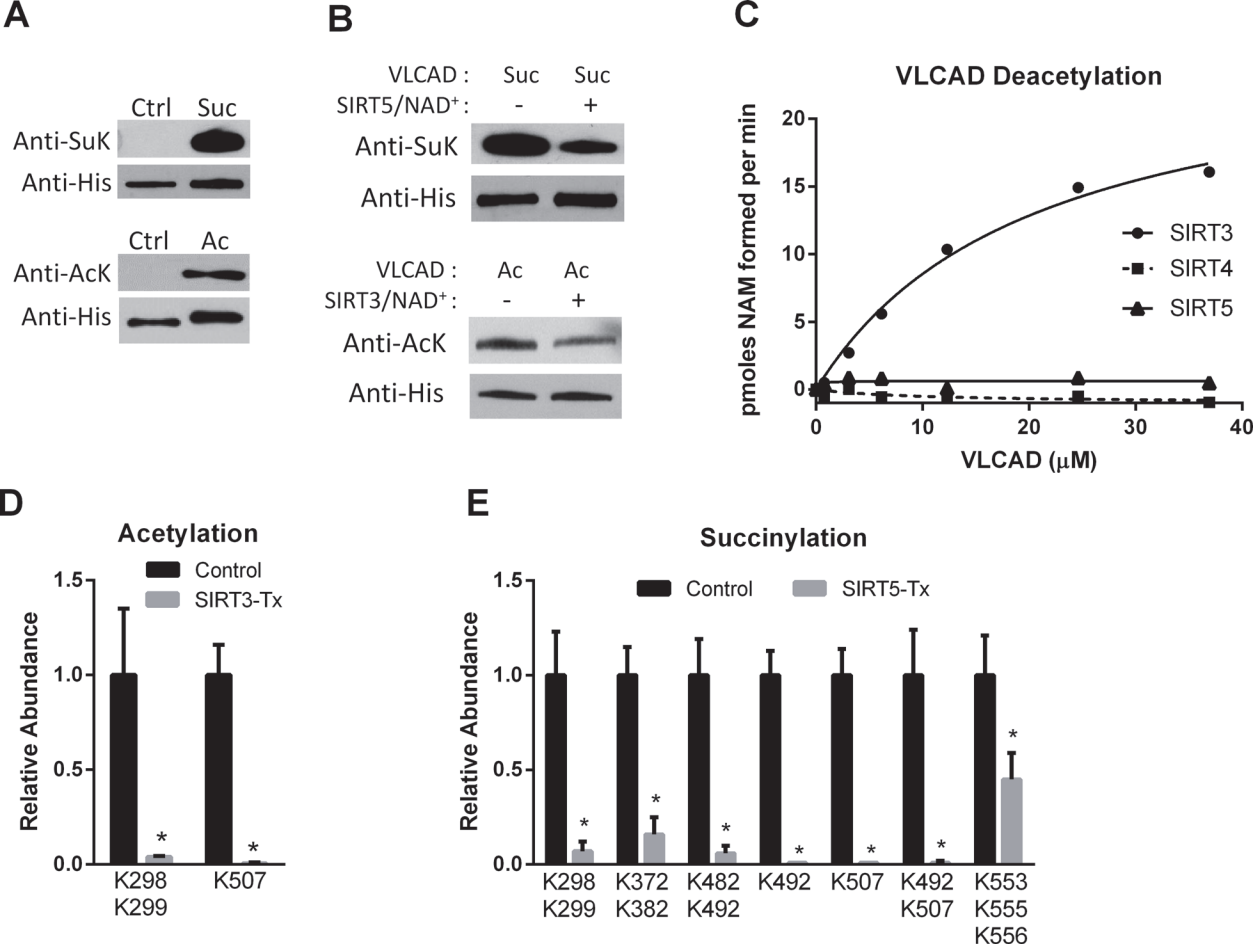
SIRT3 and SIRT5 deacylate VLCAD at overlapping sites. A) Recombinant, unmodified VLCAD (Ctrl) was subjected to chemical succinylation (top) or acetylation (bottom) which was verified by western blotting with anti-succinyllysine (SuK) or anti-acetyllysine (AcK) antibodies. B) Chemically succinylated (Suc) and acetylated (Ac) VLCAD proteins were reacted with SIRT5 and SIRT3, respectively. Changes in succinylation or acetylation were then evaluated by western blotting, with anti-His blotting as loading control. C) Only SIRT3 reacts with chemically acetylated VLCAD as determined by incubating increasing amounts of acetylated VLCAD with SIRT3, SIRT4, or SIRT5 in the presence of radiolabeled NAD+. Shown are the means of duplicate assays. D) Acetylated VLCAD was treated with SIRT3 or inactive mutant SIRT3 (Control). Quantitative mass spectrometry was used to determine the relative abundance of acetylated peptides. Shown are acetylation sites with >2-fold change. See S1 Dataset for details. E) Succinylated VLCAD was treated with SIRT5 or inactive mutant SIRT5 (Control) and succinylated peptides were quantified by mass spectrometry. Shown are succinylation sites with >2-fold change. See S2 Dataset for details. D and E both depict the means and standard deviations of quadruplicate assays.

To identify the sites of interaction between human VLCAD and the mitochondrial sirtuins we employed a strategy in which recombinant human VLCAD was chemically hyper-acetylated or chemically hyper-succinylated followed by incubation with recombinant human SIRT3 or SIRT5. The chemical modification methods produced robust acetylation and succinylation as determined by anti-acetyllysine and anti-succinyllysine western blotting (Fig. 1A). Incubation of chemically acetylated VLCAD with recombinant SIRT3 reduced the level of acetylation by almost half, while incubation of chemically succinylated VLCAD with SIRT5 reduced succinylation by ~60% (Fig. 1B). SIRT4 and SIRT5 have both been reported to have deacetylase activity but neither reacted with chemically acetylated VLCAD in a radiolabeled NAD^+^ cleavage assay (Fig. 1C), confirming the specificity of SIRT5 for succinylated VLCAD.

Next, quadruplicate samples of acetylated VLCAD and succinylated VLCAD were reacted with SIRT3 and SIRT5, respectively, followed by quantitative mass spectrometry. As controls, quadruplicate samples of chemically modified VLCAD were incubated with inactive mutant SIRT3 (H248Y) or inactive mutant SIRT5 (H158Y). The mixtures of VLCAD and sirtuins were trypsin-digested and a label-free quantitative proteomics approach [20] was used to measure the abundance of both acylated and non-acylated peptides (S1,S2 Datasets). A 2-fold change in acylation was set as the cutoff for further investigation. SIRT3 decreased the abundance of two acetylated peptides >2-fold (Fig. 1D). Peptides doubly acetylated at K298/K299 or singly acetylated at K507 were almost completely absent in the SIRT3-treated samples, suggesting that these lysines are efficiently targeted by SIRT3. SIRT5 desuccinylation of VLCAD was more extensive, with 7 different peptides showing >2-fold reductions in lysine succinylation (Fig. 1E). Like SIRT3, SIRT5 targeted K298/K299 and K507. Peptides containing succinylated K492 alone or in combination with succinylated K482 or K507 were also nearly absent in the SIRT5-treated samples, as were peptides doubly succinylated at K372/K382 or triply succinylated at K553/K555/K556.

### SIRT3/SIRT5 deacylate lysines that localize to the active site and putative membrane-binding domain of VLCAD

Mass spectrometric analysis of sirtuin-deacylated VLCAD proteins indicated that K298, K299, and K507 are SIRT3-targeted lysines and K298, K299, K372, K382, K482, K492, K507, K553, K555, and K556 are SIRT5-targeted lysines. Three-dimensional molecular modeling was used to determine how these residues might influence VLCAD function.

**Fig 2:**
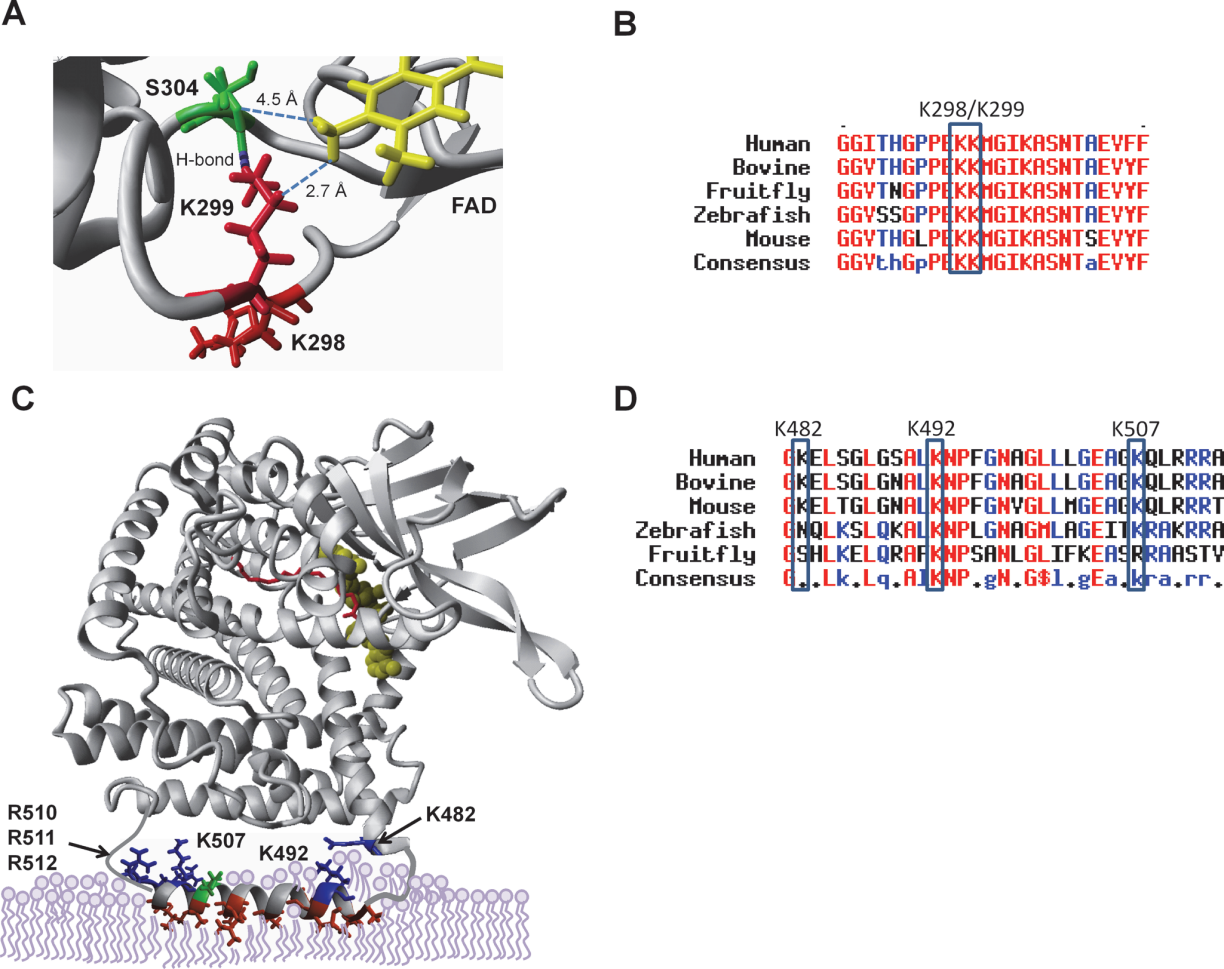
SIRT3 and SIRT5 deacylate lysines that localize to the active site and putative membrane binding domain of VLCAD. K299 (red) hydrogen bonds with neighboring S304 (green), and both are within interacting distance of the essential FAD cofactor (yellow) which is non-covalently bound in the VLCAD active site. B) Amino acid alignment of the region surrounding K299, showing conservation of this residue across diverse species. C) The portion of VLCAD spanning residues 486–518, which includes sirtuin target sites K492 and K507, is disordered in the crystal structure. PsiPred was used to generate a model of the disordered segment which was overlaid upon the structure of a VLCAD monomer. Hydrophobic residues are rendered red, positively charged residues blue, and negatively charged residues green. The active site is indicated as FAD in yellow and acyl-CoA substrate in red. D) Amino acid alignment of the putative membrane-binding amphipathic helix.

Both SIRT3 and SIRT5 efficiently targeted the peptide GFGGITHGPPE**KK**MGIK^303^ when doubly acetylated or succinylated at K298/K299. An examination of the VLCAD crystal structure reveals that K298 and K299 are located near the active site in close proximity to the FAD cofactor (Fig. 2A). These residues are highly conserved across species (Fig. 2B). While K298 does not appear to interact with other residues, K299 is predicted to hydrogen bond to S304. Both K299 and S304 are within interacting distance of the FAD cofactor. The FAD cofactor, which is absolutely required for electron abstraction during the dehydrogenation of acyl-CoA substrates, is non-covalently bound in VLCAD. From the structure it is predicted that K299 serves to coordinate FAD in the VLCAD active site.

Residue K507 was also targeted by both SIRT3 and SIRT5. Additionally, SIRT5 targeted peptides succinylated at two neighboring lysines, K482 and K492. In the VLCAD crystal structure K482 is near the end of helix K which transitions into a disordered segment of the protein that includes residues K492 and K507. The disordered region, which spans residues 486 to 518, has been hypothesized to form an amphipathic helix that mediates membrane binding [21]. We created a hypothetical model of this helix using PsiPred software and superimposed it onto the solved VLCAD structure (Fig. 2C). There are several hydrophobic residues (illustrated in red) that are predicted to bury into the phospholipids of the inner leaflet of the mitochondrial inner membrane. Five positively charged residues (K492, K507, R510, R511, and R512) and one negatively charged residue (E504) line the solvent-exposed side of the helix. K482 is predicted to orient toward this amphipathic helix from the end of helix K. Like K298/K299, these three lysines are highly conserved, particularly across mammalian species (Fig. 2D).

In addition to the residues described above, SIRT5 also appeared to target VLCAD peptides doubly succinylated at K372/K382 or triply succinylated at K553/K555/K556 (Fig. 1E). In the VLCAD crystal structure none of these five residues are near the active site or the membrane-binding domain, and therefore were not pursued further for the present studies. However, we cannot rule out the possibility that they are involved in protein:protein interactions with other members of the FAO machinery or with the electron transport chain complexes.

### The SIRT3/SIRT5 target site K299 is critical for FAD binding and VLCAD activity

**Fig 3:**
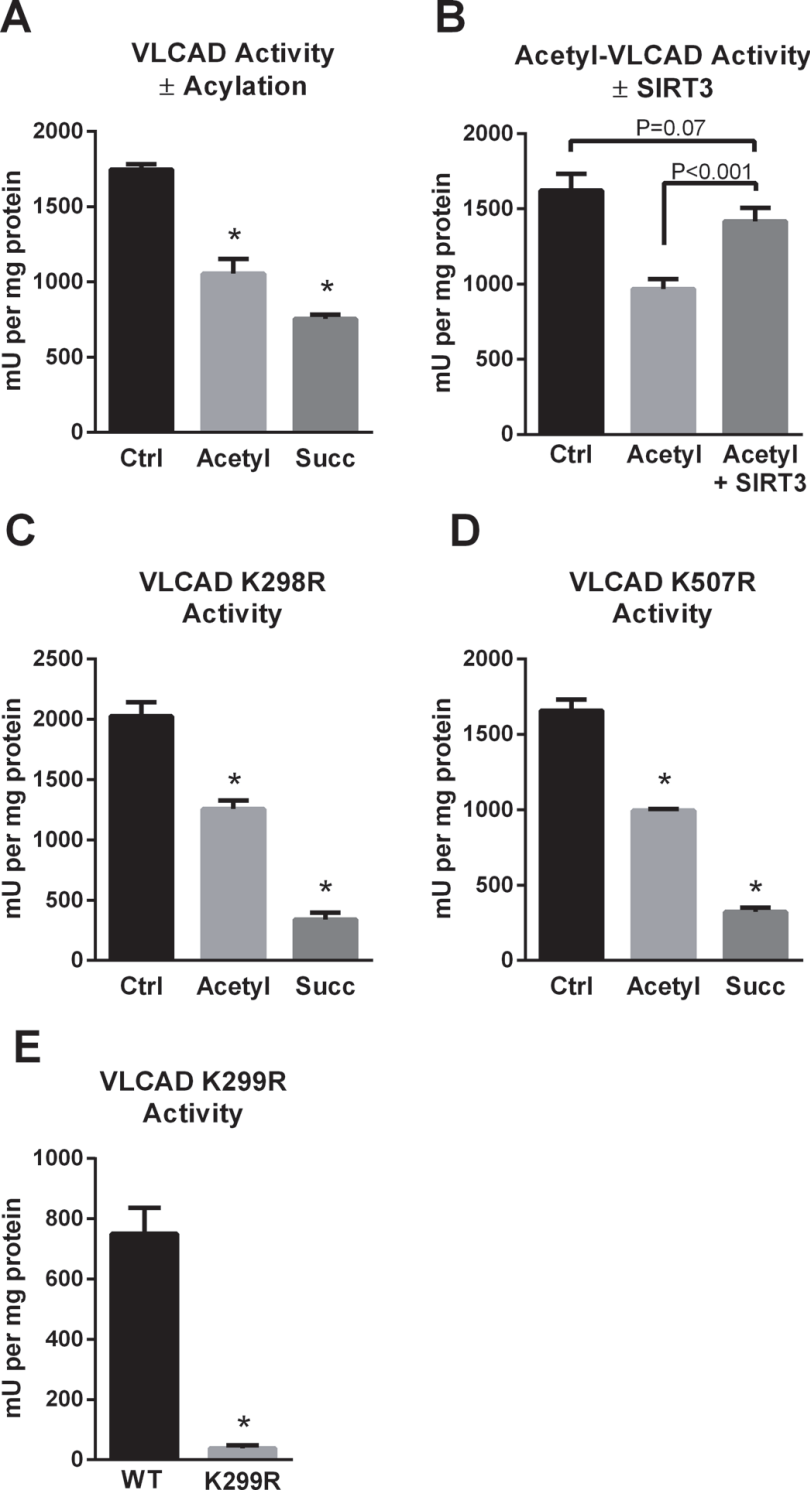
The SIRT3/SIRT5 target site K299 is critical for FAD binding and VLCAD activity. A) Chemical acetylation and succinylation both reduce enzymatic activity of recombinant VLCAD. B) Incubation of acetylated VLCAD with SIRT3 rescues activity, while incubation of succinylated VLCAD with SIRT5 does not (not shown). C) Mutant K298R retains sensitivity to acylation-induced loss of activity,suggesting that K298 does not play a mechanistic role in the reduced activity. D) Likewise, mutant K507R retains sensitivity to acylation-induced loss of activity, suggesting that K507 also does not play a mechanistic role in the reduced activity. E) K299 is highly sensitive to conservative substitution with arginine. K299R lost the yellow color characteristic of FAD and consequently became inactive. All bar graphs depict means and standard deviations of triplicate assays. *P<0.01 versus wild-type or control.

The presence of two sirtuin-targeted lysines (K298, K299) near the VLCAD active site suggested that acylation may alter enzymatic activity. Indeed, chemical acetylation or succinylation of recombinant human VLCAD was associated with a significant loss of activity with palmitoyl-CoA as substrate and ETF as the electron acceptor (Fig. 3A). The loss of activity after lysine acetylation could be recovered by incubation with SIRT3 (Fig. 3B). However, SIRT5 treatment did not recover the activity of succinylated VLCAD (data not shown). The reason for this is not clear. We postulate that the abundance of negative charges introduced on the surface of the VLCAD protein by high level chemical succinylation may repel ETF, the electron acceptor protein used in the enzyme activity assay. Charge-charge interactions are known to play a role in the binding of ETF to acyl-CoA dehydrogenase enzymes [22].

To determine which of the SIRT3-targeted lysines mediate the effect of acetylation on VLCAD activity, we introduced arginine substitutions at the SIRT3 target sites K298, K299, and K507. Arginine substitutions retain the positive charge of lysine but cannot be acylated. The arginine mutants were subjected to chemical acetylation and succinylation and tested for resistance to loss of activity. The mutants K298R and K507R retained full sensitivity to both acetylation and succinylation (Fig. 3C, D), suggesting that these two residues are not involved in the loss of activity. Unfortunately, the K299R mutant could not be tested in this way due to instability of the VLCAD protein. VLCAD K299R was poorly expressed and produced very low yields of purified protein. Moreover, the characteristic yellow color of purified VLCAD, caused by the FAD cofactor, was visibly decreased in preparations of VLCAD K299R. The ratio of absorbance at 280 nm (protein) to the absorbance at 450 nm (FAD) is an indicator of FAD content for flavoproteins such as VLCAD, with higher ratios indicating a loss of FAD. This ratio was 22.8 for VLCAD K299R versus 5.0 for wild-type VLCAD, 3.0 for VLCAD K298R, and 3.7 for K507R. Correspondingly, the K299R mutant had essentially zero enzymatic activity (Fig. 3E and could not be studied further. The close proximity of K299 to the FAD cofactor, the loss of FAD upon even a conservative arginine substitution at K299, and the sensitivity of K298R and K507R to lysine acylation suggest that K299 likely mediates the effects of acylation on VLCAD activity.

### Reversible acylation of K482, K492, and K507 regulates VLCAD binding to cardiolipin

**Fig 4:**
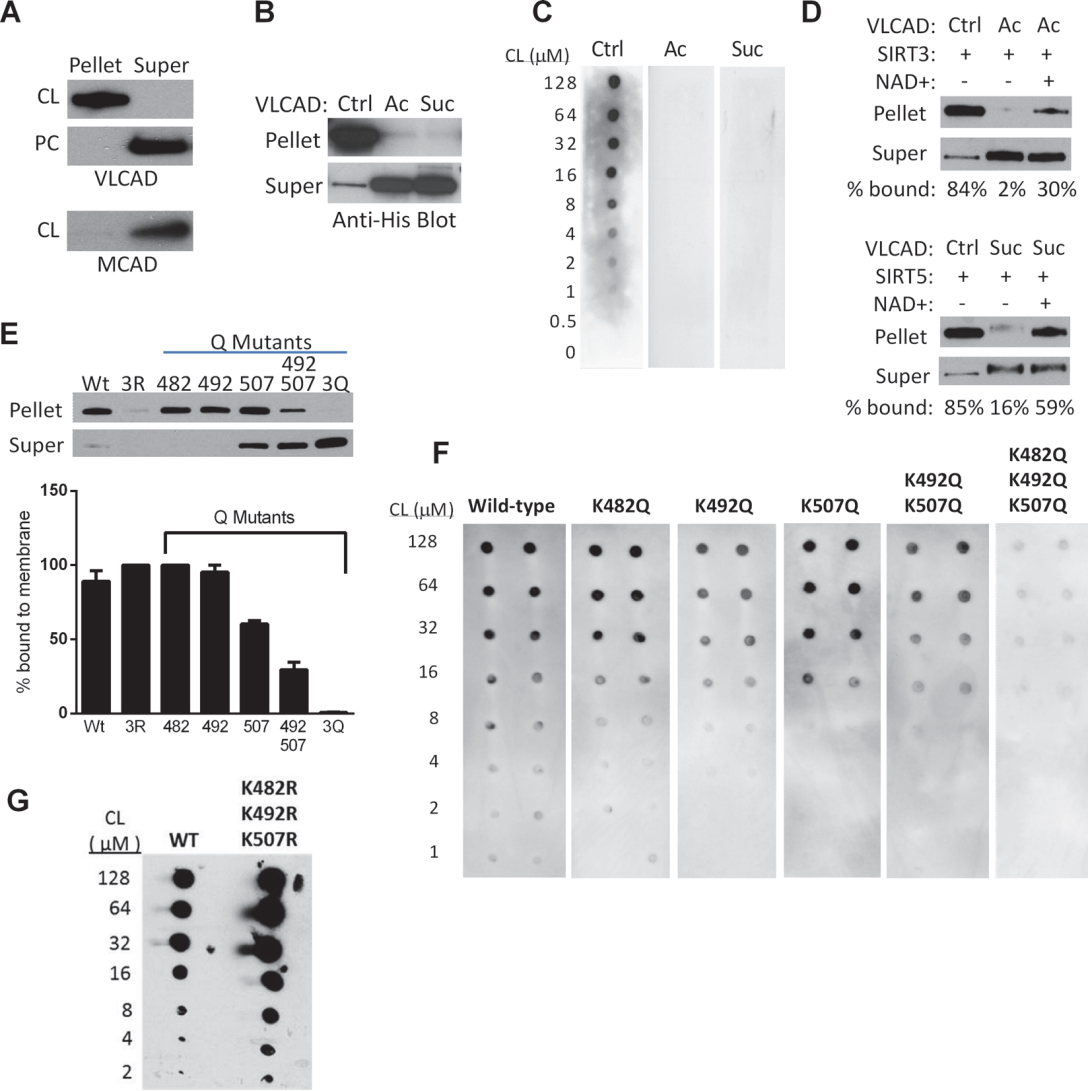
Regulation of VLCAD binding to cardiolipin by reversible acylation at K482, K492, and K507. A) Unmodified VLCAD protein is efficiently pulled down by cardiolipin (CL) containing vesicles but not by phosphatidylcholine (PC) vesicles lacking cardiolipin. MCAD, a known matrix protein, was used as control and does not bind cardiolipin. B) Unmodified (Ctrl), acetylated (Ac), and succinylated (Suc) VLCAD proteins were incubated with isolated mouse liver (VLCAD-/-) mitochondrial membranes. Acetylation and succinylation almost completely eliminated membrane binding. C) Increasing concentrations of cardiolipin (CL) were spotted onto membranes and used for a “fat blot” assay to measure lipid binding of unmodified (Ctrl), acetylated (Ac), or succinylated (Suc) recombinant VLCAD proteins. D) Top: unmodified (Ctrl) or acetylated (Ac) VLCAD were mixed with recombinant SIRT3 with or without NAD+ to induce deacetylation, in the presence of VLCAD-/- mitochondrial membranes. Densitometry was used to calculate the % signal appearing in the pellet (membrane) fraction. Bottom: the same experiment as the top panel, but with succinylated VLCAD and SIRT5. These experiments were repeated with similar results. E) The importance of charged lysines at K482, K492, and K507 was demonstrated by site-directed mutagenesis to uncharged glutamine (Q) followed by membrane-binding assays with the purified mutant proteins. 3R = triple arginine substitution, 3Q = triple glutamine substitution. Shown is a representative blot. Triplicate experiments were evaluated by densitometry and the % of protein bound to the membrane (means and standard deviations) is presented in the bar graph. F) The fat blot assay was performed to measure affinity of the same mutant proteins in panel E for cardiolipin. G) The triple arginine mutant from panel E was further studied with the fat blot method, where it showed normal or even enhanced binding to cardiolipin.

We hypothesized that the sirtuin-targeted lysines in the putative membrane-binding domain mediate binding to the mitochondrial membrane via charge-charge interactions. More specifically, we postulated an interaction between these positively charged lysines and cardiolipin, with its unique dimeric structure and two negative charges. VLCAD interaction with cardiolipin has not previously been established. We began by using cardiolipin-containing vesicles to pull down recombinant VLCAD protein. Vesicles containing a mixture of phosphatidylcholine and cardiolipin (8:1) efficiently pulled down VLCAD from solution while vesicles containing only phosphatidylcholine did not (Fig. 4A). Medium-chain acyl-CoA dehydrogenase (MCAD), known to be a matrix enzyme, was used as a control and did not bind to cardiolipin.

Acetylation of lysines neutralizes positive charges while succinylation introduces negative charges. Both acetylation and succinylation of recombinant VLCAD completely eliminated binding to isolated mitochondrial membranes (Fig. 4B). Similarly, incubation of VLCAD, acetylated VLCAD, and succinylated VLCAD with increasing amounts of cardiolipin in the “fat blot” assay indicated dose-dependent binding to cardiolipin for unmodified VLCAD and no observable binding for the acylated proteins (Fig. 4C). Incubation of acetylated VLCAD with SIRT3 and succinylated VLCAD with SIRT5 produced ~30% and ~60% rescue of membrane binding, respectively (Fig. 4D). The more robust rescue by SIRT5 may reflect the fact that SIRT5 targets all three lysines in the membrane-binding domain (K482, K492, K507) while SIRT3 targets only K507 (see Fig. 1). Site-directed mutagenesis experiments indicated that of the three lysines, K507 exerts the most influence over membrane binding. Residues K482, K492, and K507 were substituted with glutamine, which has no charge. Substitution of either K482 or K492 with glutamine had little effect on membrane binding while binding was reduced by one-third for the K507Q mutant (Fig. 4E). The double substitution K492Q/K507Q greatly reduced binding while the triple substitution K482Q/K492Q/K507Q nearly abolished it. A triple arginine mutant was also made (K482R/K492R/K507R) which was somewhat unstable but retained full membrane binding. Similar results were obtained using the fat blot method to measure binding of the VLCAD glutamine mutants to cardiolipin (Fig. 4F).

**Fig 5:**
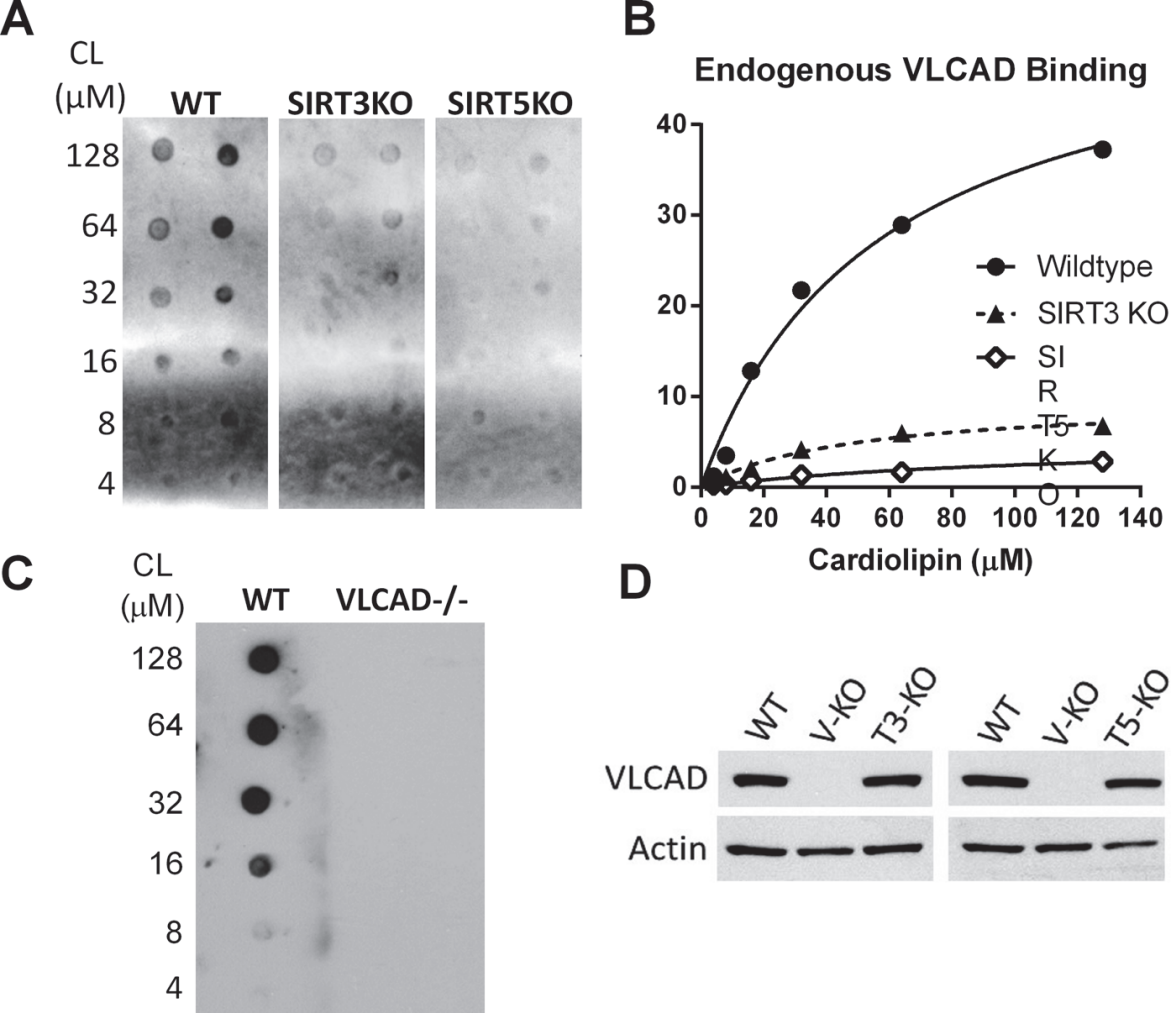
VLCAD from SIRT3 and SIRT5 knockout mice shows reduced affinity for cardiolipin. A) The fat blot method was used to evaluate endogenous VLCAD binding to cardiolipin in fasted (20 hr) mouse liver lysates. B) Densitometry was used to quantify binding from panel A. C) Lysate from VLCAD-/- liver was tested as a negative control and shows no detectable signal. D) Western blot was used to confirm that total VLCAD expression is not significantly different between wild-type, SIRT3 KO, and SIRT5 KO mice.

Based on these results, we hypothesized that VLCAD would show impaired membrane binding in SIRT3 and particularly in SIRT5 knockout mice. To test this, increasing concentrations of cardiolipin (4 to 128 μM) were spotted onto nitrocellulose membranes and incubated with 500 μg of total liver lysate from either wild-type, SIRT3-/-, or SIRT5-/- mice. The resulting cardiolipin-bound VLCAD was visualized by anti-VLCAD antibody and quantified by densitometry. Sirtuin knockout mice showed less cardiolipin-bound VLCAD despite having comparable total VLCAD antigen levels in liver homogenates (Fig. 5).

## Discussion

VLCAD, a homodimeric flavoprotein that is part of the acyl-CoA dehydrogenase family, is an essential long-chain FAO enzyme in human heart, muscle, and liver. It functions in the context of a multi-protein complex that contains other FAO proteins and interacts with mitochondrial respiratory chain supercomplexes [18]. Here, we have shown that lysine acylation negatively affects VLCAD function. This may occur by directly disturbing the active site (acylation at K299) or by disrupting its ability to interact with cardiolipin (acylation at K482, K492, K507), or both. If VLCAD does not properly localize to the inner mitochondrial membrane then flux through the FAO pathway will be reduced. This is evidenced by patient mutations in the domain that we now show to be critical for binding to cardiolipin, which encompasses residues 486–516. We previously evaluated patient mutations A490P and L502P [13]. As recombinant proteins, both of these patient mutations exhibited higher activity than wild-type VLCAD, likely because the mutations stabilized the disordered membrane-binding domain making the enzyme more stable in solution. Yet, despite the higher enzymatic activity, patients with these mutations experience symptoms because the VLCAD protein does not bind to the membrane and thus becomes mis-localized [13].

VLCAD’s obligate partners in FAO are CPT2 and TFP. Together, these three enzymes can chain-shorten long-chain fatty acids down to medium-chain fatty acids. All three localize to the inner mitochondrial membrane. CPT2 and TFP have both been shown to interact with cardiolipin [14, 15] and now we show similar results for VLCAD. Cardiolipin is important for the formation of high molecular weight “supercomplexes” in the electron transport chain [17], and may also be involved in recruiting the putative FAO complex. Acylation of the VLCAD cardiolipin-binding helix at K482, K492, and/or K507 could reduce function of this higher-order metabolic complex. We speculate that when the local concentration of the metabolites acetyl-CoA or succinyl-CoA become high, lysine acylation of VLCAD will increase, resulting in subsequent feedback inhibition of FAO by promoting for disassembly of the FAO complex. Induction of SIRT3 and SIRT5 activity by starvation or by rising NAD^+^ levels would cause VLCAD deacylation and recruitment of the VLCAD protein back to the mitochondrial membrane. Similar mechanisms may apply to CPT2 and TFP.

The SIRT3 and SIRT5 target sites we have identified on human VLCAD overlap, with both sirtuins deacylating K298/K299 and K507. Currently, the mechanisms that regulate lysine acylation in the mitochondria are not understood. Acetylation of mitochondrial proteins has been suggested to be enzyme-catalyzed [23], but may also occur by non-enzymatic mechanisms [24]. No lysine succinyltransferase enzyme has been identified. Thus, the significance of the overlapping SIRT3/SIRT5 target sites is not clear. The overlap may represent crosstalk between two regulatory pathways, or these residues may simply be chemical modification “hotspots” that are prone to modification by acyl-CoA metabolites. Interestingly, although the role of ubiquitin modifications on intra-mitochondrial proteins is not understood, human VLCAD has been shown to be ubiquitinated on K492 and K507 [25]. It is possible that acylation at these sites extends the half-life of the protein by blocking ubiquitination, essentially creating a reserve pool of VLCAD that can be re-activated upon demand. Further work is now necessary to elucidate the relative stoichiometries of these modifications, possible inter-sirtuin crosstalk, and crosstalk with other post-translational modifications such as ubiquitination.

## Supporting Information

S1 Dataset Deacetylation of chemically acetylated recombinant VLCAD by SIRT3. (XLSX)

S2 Dataset Desuccinylation of chemically succinylated recombinant VLCAD by SIRT5. (XLSX)
